# Delayed necrostatin-1s infusion attenuates cystic white matter injury in preterm fetal sheep

**DOI:** 10.1016/j.neurot.2025.e00775

**Published:** 2025-11-07

**Authors:** Benjamin A. Lear, Christopher A. Lear, Simerdeep K. Dhillon, Victoria J. King, Justin M. Dean, Joanne O. Davidson, Alistair J. Gunn, Laura Bennet

**Affiliations:** The Fetal Physiology and Neuroscience Group, Department of Physiology, The University of Auckland, Auckland, New Zealand

**Keywords:** Hypoxia-ischaemia, Periventricular leucomalacia, Preterm, Necrostatin-1s, White matter injury

## Abstract

Severe, cystic white matter injury (WMI) in preterm infants is associated with neurodevelopmental impairment. Strikingly, it often develops weeks after birth. In the present study we tested the hypothesis that necrotic programmed cell death, necroptosis, may be a key mediator of severe WMI, using the specific inhibitor necrostatin-1s (Nec-1s). Chronically instrumented preterm fetal sheep (0.7 gestation) received 25 ​min of hypoxia-ischaemia induced by complete umbilical cord occlusion (UCO) or sham-UCO (controls, *n* ​= ​9), followed by intracerebroventricular infusion of Necrostatin-1s at 3, 8 and 13 days after UCO (UCO-Nec-1s, *n* ​= ​8) or vehicle (UCO-vehicle, *n* ​= ​9). Histology was obtained at 21 days after UCO. UCO-vehicle was associated with a spectrum of brain injury including diffuse WMI, and in 7/9 fetuses, generalised white matter atrophy, ventriculomegaly and/or temporal lobe cystic lesions. Necrostatin-1s infusion was associated with less severe WMI in the temporal lobe (1/8, *p* ​= ​0.041), with reduced atrophy, increased density of mature oligodendrocyte cells, myelin area fraction, and reduced microgliosis but increased numbers of apoptotic cells. Interestingly, diffuse white matter injury in the parietal tracts was not affected. This study suggests that delayed, severe white matter injury after acute hypoxia-ischaemia is mediated by slowly evolving necroptosis and neuroinflammation. We speculate that Nec-1s shifts the dominant cell death pathway from necrosis toward apoptosis, allowing reduced inflammation and so preserving oligodendrocyte populations and myelination in the temporal lobe. These results suggest that necroptosis is a promising therapeutic target to mitigate severe white matter injury.

## Introduction

Preterm infants are highly susceptible to white matter injury (WMI). It is well established that severe, cystic WMI is associated with increased risk of subsequent cerebral palsy [1,2], and strikingly, it often develops on ultrasonography at a median of ∼4 weeks after birth [3,4]. We recently demonstrated in preterm fetal sheep that hypoxia-ischaemia (HI) induced by complete UCO for 25 ​min is associated with early selective diffuse WMI, as shown by the loss of mature oligodendrocytes and reduced myelination [5], followed by progression to severe WMI after 14–21 days recovery. This very delayed WMI was characterised by bilateral cystic lesion formation in the temporal lobe white matter, ventriculomegaly and white matter atrophy [5]. Interestingly, the location of cystic WMI appeared to be closely related to the location of large microglial aggregates at earlier time points, suggesting that exuberant neuroinflammation was a key factor the progression of severe, cystic WMI. Supporting this hypothesis, in this model delayed intracerebroventricular (ICV) infusions of a selective tumor necrosis factor (TNF) antagonist, Etanercept, starting 3 days after injury ameliorated severe WMI [6].

A key mechanism mediating this slowly evolving severe WMI is likely to be programmed cell death through necrotic pathways (necroptosis) [7,8]. Necroptosis is initiated by the activation of membrane-bound death receptors via TNF family cytokines [9]. Necroptosis requires inhibition of caspase processes and/or mitochondrial failure, which blocks the typical apoptosis pathway triggered by membrane-bound death receptor activation, such as TNFR1 and TNFR2 [10]. Activation of death receptors leads to the deubiquitination of the receptor-interacting protein (RIP) kinases RIP1 and RIP3, allowing RIP1 and RIP3 to form a necrosome which activates the mixed lineage kinase domain-like protein (MLKL). MLKL forms pores within the cell and organelle membranes, leading to permeabilization. Oncosis then occurs as the cell swells, eventually resulting in cellular lysis with a necrotic phenotype [11]. Given that activation of RIP1 is preceded by TNF binding to TNFR1 and TNFR2 [12], it is plausible that the effect of Etanercept to mitigate severe macroscopic WMI was ultimately mediated by blockade of necroptosis [6].

Nec-1 is a small molecule that specifically inhibits the pro-necroptotic protein RIP1, preventing formation of the necrosome. In adult mice exposed to middle cerebral artery occlusion, ICV infusion of Nec-1 significantly reduced infarct volumes [13]. In neonatal postnatal day (P)7 mice exposed to acute HI, Nec-1 administered 15 ​min after the end of hypoxia reduced infarct volume and ameliorated oxidative damage and inflammation at P11 and P28 [8]. Interestingly, Nec-1 also attenuated mitochondrial failure in the secondary phase of HI injury [14]. However, it is unknown whether delayed RIP1 blockade, started after the resolution of secondary events, would mitigate late-evolving WMI.

Therefore, in the present study, we tested the hypothesis that delayed RIP1 blockade with Necrostatin-1 stable (Nec-1s) started 3 days after HI in preterm fetal sheep at 104–105 days of gestation, broadly equivalent to 28–32 weeks human gestation [15], would ameliorate severe WMI, including cystic WMI, after 3 weeks recovery [6].

## Materials and Methods

All procedures were approved by the Animal Ethics Committee of the University of Auckland and were carried out in accordance with the Animal Welfare Act of New Zealand 1999 and the University of Auckland’s Code of Ethical Conduct for the use of animals for teaching and research, approved by the Ministry of Primary Industries, Government of New Zealand. This manuscript is compliant with the ARRIVE guidelines for reported animal research [16].

### Subjects and surgical procedures

This study was conducted in 28 time-mated Romney-Suffolk cross ewes at 2–3 years of age with previous successful pregnancy experience. On the farm, pregnancy and parity were confirmed with ultrasound. Ewes were acclimatised to concentrated pellet feed (Dunstan Nutrition, Hamilton, New Zealand) for two weeks before arrival at the laboratory. Trained staff assessed the ewes’ health status before acceptance into the laboratory. All ewes were housed together in separate metabolic crates and allowed to acclimatise to the laboratory environment for one week. Throughout the entire stay, ewes had *ad-libitum* access to food and water, the rooms were climate controlled (ambient temperature: 16 ​± ​1.0 ​°C; humidity: 50 ​± ​10 ​%), and there was a 12:12 ​h light and dark cycle (light at 6 a.m.). Any sheep who did not adapt or were deemed unfit for surgery were returned to the farm.

Twenty-eight fetuses were surgically instrumented at 98–101 gestational days (term is 147 days) as previously described [6]. Ewes were given long acting oxytetracycline (20 ​mg/kg, Phoenix Pharm Distributors, Auckland, New Zealand) intramuscularly 30 ​min before surgery for antibiotic prophylaxis. Anesthesia was induced via intravenous propofol (5 ​mg/kg; AstraZeneca, Auckland, New Zealand) and intubated with an appropriately sized cuffed inflatable endotracheal tube. Anesthesia was maintained by 2–3 ​% isoflurane (Lunan Better Pharmaceutical Co.) in oxygen from a CIG Midget 3 anesthetic apparatus (Commonwealth Industrial Gases, Melbourne, VIC, Australia). Ewes received a constant infusion of isotonic saline (approximately 250 ​mL/h) to maintain fluid balance. Throughout the surgery, train anesthetic staff constantly monitored the depth of anesthesia and ewe’s vitals, including pulse rate and saturation, using a Biolight 8500 patient monitor (Guangdong Biolight Meditech co., Zhuhai, China). The surgical field was prepared by shearing the ewe’s abdomen, then washing the skin with soap and water, 2 ​% chlorohexidine gluconate solution (Onelink, Auckland, New Zealand) and 10 ​% povidone solution (F.H. Faulding and Co., Mulgrave, Victoria, Australia). A surgical team scrubbed, and donned sterile gloves and gowns before covering the ewe with sterile drapes. All the surgical procedures were conducted using strict aseptic techniques.

A midline abdominal incision was made to expose the uterus, and the fetus was partially exteriorised for instrumentation. Polyvinyl catheters (SteriHealth, Dandenong South, VIC, Australia) were placed in the left saphenous artery to measure arterial blood pressure and the right brachial artery for pre-ductal blood sampling. An additional catheter was placed into the amniotic sac for measurement of pressure within the amniotic space. Two electrodes (AS633-5SSF; Cooner Wire, Chatsworth, CA, USA) were placed subcutaneously over the right shoulder and at the level of the left fifth intercostal space to measure the fetal electrocardiogram (ECG). An intracerebroventricular (icv) catheter was placed into the right lateral ventricle (6 ​mm anterior and 4 ​mm lateral to bregma). Finally, an inflatable silicone occluder was placed around the umbilical cord to facilitate umbilical cord occlusions (OC18HD; In Vivo Metric, Healdsburg, CA, USA). Gentamicin was administered into the amniotic sac (80 ​mg; Pfizer, Auckland, New Zealand) before the uterus was closed. The maternal midline skin incision was infiltrated with 10 ​mL 0.5 ​% bupivacaine plus adrenaline (AstraZeneca) to provide long-acting analgesia. All fetal leads were exteriorised through the maternal flank, and a maternal long saphenous vein was catheterised for post-operative care and euthanasia.

Following surgery, the ewe was revived from anesthesia, extubated, and shifted into a metabolic crate. All ewes were monitored by researchers and staff, supported by a veterinary team, for signs of distress such as reduced appetite or thirst, bruxism, lethargy, and excessive vocalisations. Ewes were housed together in separate metabolic cages with *ad libitum* access to food and water and given daily intravenous antibiotics (600 ​mg benzylpenicillin sodium, Novartis, Auckland, New Zealand; 80 ​mg gentamicin) for 4 days after surgery. Fetal catheters were maintained patent with continuous infusion of heparinised saline (20 U/mL at 0.2 ​mL/h).

### Data acquisition and recordings

Fetal mean arterial pressure (MAP) and ECG were recorded continuously from 24 ​h before umbilical cord occlusion (UCO) [17]. All signals were processed and initially digitised at a sampling rate of 4096 ​Hz before being decimated to lower sampling rates and stored using customised LabVIEW-based data acquisition software (National Instruments, Austin, TX, USA). Fetal arterial blood pressure was recorded using Novatrans III Gold, MX860 pressure transducers (Medex, Hilliard, OH, USA) and corrected for maternal position by subtraction of amniotic fluid pressure. The pressure signals were amplified 500 ​× ​, low-pass filtered with a fifth-order Butterworth filter set at 20 ​Hz and saved at 64 ​Hz. The raw ECG signal was analogue filtered with a first-order high-pass filter set at 1 ​Hz and an eighth-order low-pass Bessel filter set at 100 ​Hz and saved at 1024 ​Hz.

### Experimental protocol

Experiments began at 9:00 a.m., 4−6 days after surgery (104−105 days of gestation). Fetuses were randomly assigned to either Sham-UCO (*n* ​= ​9 [*n* ​= ​5 male, *n* ​= ​4 female]), UCO-Vehicle (*n* ​= ​9 [*n* ​= ​3 male, *n* ​= ​6 female], or UCO-Nec-1s (*N* ​= ​8 [*n* ​= ​6 male, *n* ​= ​2 female]) to receive HI induced by complete UCO for 25 ​min. Fetal arterial blood samples (0.3 ​mL) were taken before the start of the experiment, at 5 and 17 ​min during occlusion, and at 2, 4, and 6 ​h after occlusion, then daily thereafter between 8:30 and 9:30 a.m. Blood samples were analyzed for pH and blood gases (ABL 800; Radiometer, Copenhagen, Denmark) and glucose and lactate levels (YSI model 2300; Yellow Springs, OH, USA). Selected timepoints from 1 to 21 days after UCO are presented for brevity.

### Drug preparation and infusion

Nec-1s was selected for this study because of its greater specificity and stability compared with Nec-1 [18,19]. Moreover, it is not associated with offsite inhibition of indoleamine 2,3-dioxygenase, an immune regulator that controls the rate of flux between the pathways leading to pro- or anti-inflammatory cytokine production [19,20]. The dosage of Nec-1s was based on that of 0.1 ​μL of 80 ​μM/L administered ICV in P7 mice [8] and extrapolated based on the mean brain weight of 40 ​g in our studies at this gestational age. Nec-1s was infused at a concentration of 0.682 ​μg/mL, dissolved in 0.5 ​mL artificial cerebrospinal fluid (aCSF) [6]. Equal molar Ɣ-cyclodextrin to Nec-1s was dissolved in the aCSF to improve the solubility of Nec-1s.

Two days after UCO and 17 ​h before the start of drug infusion, the ICV line dead space was cleared slowly at a rate of less than 50 ​μL/h aCSF. At 3 days post-UCO (when the drug reached the lateral ventricle), the infusion rate was increased to 150 ​μL/h to deliver 600 ​μL over 4 ​h. After 4 ​h, the infusion rate was decreased to 20 ​μL/h to deliver an additional 380 ​μL to completely clear the line of Nec-1s before the infusion was ended. This process was repeated with fresh Nec-1s and aCSF at day 8 and day 13 post-UCO. These timings were selected to replicate the previous study that utilised for ICV Etanercept infusions [6].

The ewes and fetuses were killed 21 days after UCO by an overdose of sodium pentobarbitone given intravenously to the ewe (9 ​g Pentobarb 300; Provet New Zealand, Auckland, New Zealand). This method is consistent with the Animal Welfare Act of New Zealand.

### Histological preparation

**Fig. 1:**
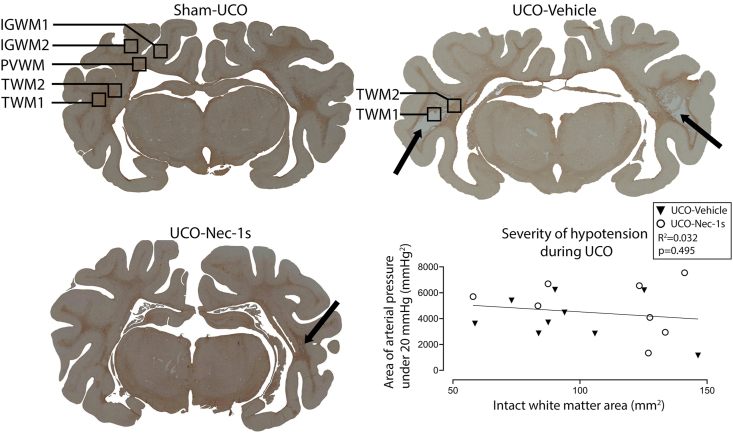
**Regions of interest and severity of hypotension**. This illustration shows examples of coronal sections stained for Iba-1-positive microglia from each group and region of interest. The black arrows highlight areas of cystic injury. Images were taken at 2.5x magnification. Note the small cystic lesion in the right hemisphere of the UCO-Nec-1s section. The graph demonstrates no relationship between the severity of hypotension during UCO (assessed as the area of arterial pressure under 20 ​mmHg) and the intact white matter area.

At post-mortem, fetal brains were perfusion fixed *in situ* with 10 ​% phosphate-buffered formalin and remained in formalin for 1 week before being processed and paraffin embedded. 10 ​μm thick coronal sections were cut using a rotary microtome (RM2235, Leica Microsystems, Wetzlar, Germany). Sections were taken 17 ​mm anterior to stereotaxic zero to examine the white matter of the parietal and temporal lobes (Fig. 1) [21]. Slides were dewaxed in xylene, rehydrated in decreasing concentrations of ethanol and subsequently washed in 0.1 ​mol/L phosphate buffered saline. To examine macroscopic structural integrity two sections per animal were stained with both thionine (Scharlau, Barcelona, Spain) and acid fuchsin (Sigma-Aldrich, Sydney, NSW, Australia) and then dehydrated in increasing concentrations of alcohol followed by xylene and mounted with coverslips.

Additional sections (two sections per animal) were labeled for immunohistochemistry. Following dewaxing and rehydration as above, antigen retrieval was performed in citrate buffer with the 2100 Antigen Retriever (Aptum Biologics, Southampton, UK). Endogenous peroxidase was quenched by incubation in 1 ​% H_2_O_2_ in methanol (or phosphate-buffered saline for Olig-2). Blocking was performed for 1 ​h at room temperature in 3 ​% normal goat serum (NGS). Sections were labeled with monoclonal primary antibodies all at 1:200 concentration: rabbit anti-oligodendrocyte transcription factor 2 (Olig-2; Abcam, AB177487), mouse anti-cleaved caspase-3 (Cell Signals, Boston, MA, USA, #9661), mouse anti-adenomatous polyposis coli clone CC1 (CC1; Merk-Millipore, OP80-100UG), mouse anti-2′,3′-cyclic-nucleotide 3′-phosphodiesterase (CNPase; Abcam, AB631), rabbit anti-myelin basic protein (MBP, Merck-Millipore, MAB381), goat anti-ionised calcium-binding adapter molecule 1 (Iba-1; Abcam, AB178846) and rabbit anti-glial fibrillary acidic protein (GFAP; Abcam, AB68428) overnight at 4 ​°C. Sections were incubated for 3 ​h with the species appropriate biotin-conjugated monoclonal secondary antibody (Vector Laboratories, Burlingame, CA, USA, BA-9200 or BA-1000) at 1:200 dilution in 3 ​% NGS. Slides were incubated in ExtrAvidin (Sigma-Aldrich) at a dilution of 1:200 in NGS for 2 ​h and then reacted with diaminobenzidine tetrachloride (Sigma-Aldrich). The reaction was halted by immersion in phosphate-buffered saline. Slides were dehydrated in increasing concentrations of alcohol followed by xylene and mounted with coverslips.

### White matter and cystic lesion area analysis

Macroscopic examination of the structural integrity of the parietal and temporal lobe white matter tracts was performed on sections stained with acid fuchsin/thionine by a blinded assessor. Blinding across the study was achieved by independent coding of slides and data files. Macroscopic examination was performed on whole section images taken on a Zeiss Axio Imager Z2 microscope with an automated motorised stage (Carl Zeiss AG, Oberkochen, Germany). Serial images were collected at 2.5 ​× ​magnification and collated using VSlide stitching software (MetaSystems, Altlussheim, Germany). Total cortical, hippocampal, lateral ventricle and white matter areas were measured using ImageJ software (National Institute of Health, Bethesda, MD, USA). The cortical area included combined measurements from the parietal and temporal lobes. The total white matter area included all continuous white matter tracts of both the parietal and temporal lobes, including the intragyral and periventricular regions and the corpus callosum. Macroscopic cystic WMI was identified in the UCO groups, most commonly in the temporal lobe, as previously described [5]. The area of each identified white matter lesion was measured and subtracted from the total white matter area to give a final intact white matter area. Although all care was taken to preserve tissue integrity and sections for area measurements were obtained at comparable anatomical levels ventricular measurements on histological sections are limited by potential tissue distortion and variability in the plane of section.

### Parietal and temporal lobe microscopy

Images of Olig2-, CC1-, CNPase-, MBP-, Iba-1-, cleaved caspase-3- and GFAP- labeled tissues were taken from two regions in the temporal lobe and three regions in the parietal lobe (Fig. 1) at 20 ​× ​on an Eclipse 80i microscope (Nikon Corporation). These five regions were imaged on both the left and right cerebral hemispheres (Fig. 1). In the temporal lobe, the first image (temporal white matter 1, TWM1) was taken from the center of the temporal lobe white matter — this represents the region with the most severe injury at 21 days after UCO, which includes the appearance of cystic white matter lesions [5]. The second image (TWM2) was taken from the superomedial white matter of the temporal lobe, which represents a less severely injured region [5]. The temporal lobe cystic white matter lesions were surrounded by a border of Iba-1-positive and GFAP-positive cells, so TWM2 was positioned outside of this border to ensure that it was in a less severely injured region and did not include the dense cellular border. Images from representative regions were taken in animals that had no identifiable cystic lesions, which included all control animals. In the parietal lobe, images were taken from the first and second intragyral white matter (IGWM1, IGWM2) and the periventricular white matter (PVWM) regions (Fig. 1).

Cell counts for total oligodendrocytes (Olig2-positve cells), mature oligodendrocytes (CC1-positive cells), apoptotic cells (cleaved caspase-3-positive cells), microglia (Iba-1 positive cells, which includes almost all cells of the monocyte/macrophage lineage [22]) and astrocytes (GFAP-positive cells) were performed by manual counts using ImageJ (National Institute of Health). Images from both left and right hemispheres were counted and averaged. Microglia showing either an ameboid or ramified morphology were included. The area fractions of MBP-positive and CNPase-positive labeling were quantified using ImageJ (National Institute of Health) [5,6].

### Statistical analysis

Statistical analysis was performed using SPSS v28 (SPSS, Chicago, IL, USA). The Shapiro-Wilks test was used to assess normality. Histological outcomes in the parietal lobe regions (IGWM1, IGWM2 and PVWM) were assessed using two-way ANOVA with group as the independent factor and area treated as repeated measures. The temporal lobe regions were assessed separately using one-way ANOVA with the group as the independent factor. Analysis of post-mortem and biochemistry data were assessed by one-way ANOVA with group as the independent factor. If a significant group effect was found in the above analyses, the Tukey post-hoc test was additionally performed. Incidences were assessed using the Fisher Exact test. Statistical significance was accepted when *p* ​< ​0.05. Data are mean ​± ​SEM.

## Results

### Biochemistry, umbilical cord occlusion and post-mortem results

All fetuses were healthy, with normal physiological and blood gas parameters based on our laboratory standards before the start of experiments. There were no significant differences in any physiological parameters between the groups before the experiments. UCO was associated with sustained bradycardia and profound arterial hypotension, hypoxemia, hypoglycaemia, and progressive respiratory and metabolic acidosis (Table 1). A significant difference in lactate was observed between the UCO-Nec-1s and UCO-Vehicle groups at 17 ​min during UCO.

**Table 1:** Fetal pH, blood gases and metabolites.

	Group	Baseline	UCO (5 ​min)	UCO (17 ​min)	+2 ​h	+4 ​h	+6 ​h	+1 day	+3 days	+7 days	+14 days	+21 days
pH	Sham-UCO	7.36 ​± ​0.01	7.36 ​± ​0.01	7.36 ​± ​0.01	7.36 ​± ​0.01	7.35 ​± ​0.01	7.35 ​± ​.01	7.35 ​± ​0.01	7.34 ​± ​0.01	7.35 ​± ​0.01	7.34 ​± ​0.01	7.33 ​± ​0.01
	UCO-vehicle	7.35 ​± ​0.01	7.03 ​± ​0.01^a^	6.84 ​± ​0.01^a^	7.29 ​± ​0.02^a^	7.38 ​± ​0.01	7.40 ​± ​0.01^a^	7.36 ​± ​0.01	7.36 ​± ​0.01	7.36 ​± ​0.01	7.35 ​± ​0.01	7.36 ​± ​0.01
	UCO-Nec-1s	7.35 ​± ​0.01	7.03 ​± ​0.01^a^	6.84 ​± ​0.02^a^	7.29 ​± ​0.02^a^	7.37 ​± ​0.01	7.39 ​± ​0.01^a^	7.36 ​± ​0.01	7.36 ​± ​0.01	7.36 ​± ​0.01	7.35 ​± ​0.01	7.34 ​± ​0.02
p_a_CO_2_ (mmHg)	Sham-UCO	48.5 ​± ​0.7	47.1 ​± ​0.8	46.6 ​± ​0.8	47.3 ​± ​0.9	46.1 ​± ​1.1	46.7 ​± ​0.9	47.1 ​± ​0.7	48.4 ​± ​0.8	49.1 ​± ​0.8	48.6 ​± ​1.0	49.4 ​± ​1.2
	UCO-vehicle	50.7 ​± ​1.4	99.7 ​± ​2.6^a^	136.4 ​± ​6.0^a^	47.8 ​± ​0.8	47.7 ​± ​1.1	48.5 ​± ​0.7	46.3 ​± ​1.4	46.8 ​± ​1.5	49.2 ​± ​1.8	50.7 ​± ​1.4	48.6 ​± ​1.1
	UCO-Nec-1s	50.6 ​± ​1.2	92.7 ​± ​3.7^a^	118.2 ​± ​8.5^a^	48.7 ​± ​1.5	47.1 ​± ​1.0	46.0 ​± ​0.8	46.6 ​± ​1.1	48.2 ​± ​1.1	48.8 ​± ​0.8	48.2 ​± ​1.5	48.7 ​± ​2.3
p_a_O_2_ (mmHg)	Sham-UCO	25.6 ​± ​0.6	24.6 ​± ​0.7	24.3 ​± ​0.6	25.5 ​± ​0.7	24.9 ​± ​0.5	25.7 ​± ​0.7	26.2 ​± ​0.8	27.0 ​± ​0.7	25.3 ​± ​1.1	26.0 ​± ​1.4	22.6 ​± ​1.3
	UCO-vehicle	24.7 ​± ​0.8	6.6 ​± ​0.4^a^	9.5 ​± ​1.2^a^	27.2 ​± ​1.2	23.5 ​± ​1.0	25.4 ​± ​1.0	29.1 ​± ​1.0^a^	31.1 ​± ​1.2^a^	29.4 ​± ​1.5	28.4 ​± ​1.2	26.5 ​± ​1.8
	UCO-Nec-1s	25.3 ​± ​0.9	5.3 ​± ​0.8^a^	6.4 ​± ​1.4^a^	27.4 ​± ​0.8	25.1 ​± ​1.7	26.6 ​± ​1.6	28.2 ​± ​0.7	30.4 ​± ​1.3	27.5 ​± ​1.4	28.1 ​± ​1.5	27.0 ​± ​1.4
Hct (%)	Sham-UCO	27.2 ​± ​0.7	26.2 ​± ​0.8	26.1 ​± ​0.9	26.7 ​± ​0.7	28.2 ​± ​0.2	25.9 ​± ​0.9	26.9 ​± ​0.8	29.4 ​± ​1.4	31.6 ​± ​1.8	31.3 ​± ​1.3	31.4 ​± ​0.8
	UCO-vehicle	26.2 ​± ​0.7	27.8 ​± ​1.1	28.2 ​± ​0.8	28.1 ​± ​0.7	27.4 ​± ​0.5	27.7 ​± ​0.8	29.0 ​± ​0.5	30.2 ​± ​2.2	33.6 ​± ​3.7	35.2 ​± ​4.4	30.2 ​± ​0.3
	UCO-Nec-1s	26.3 ​± ​0.9	27.4 ​± ​0.8	27.1 ​± ​1.5	29.2 ​± ​1.1	27.7 ​± ​0.5	27.4 ​± ​1.0	29.0 ​± ​1.3	28.4 ​± ​1.3	32.9 ​± ​3.1	35.8 ​± ​3.5	35.2 ​± ​3.1
O_2_ct (mmol/L)	Sham-UCO	3.7 ​± ​0.1	3.4 ​± ​0.1	3.3 ​± ​0.2	3.6 ​± ​0.1	3.2 ​± ​0.1	3.4 ​± ​0.2	3.7 ​± ​0.2	3.9 ​± ​0.1	3.9 ​± ​0.2	6.6 ​± ​2.7	3.3 ​± ​0.3
	UCO-vehicle	3.6 ​± ​0.1	0.4 ​± ​0.1^a^	0.5 ​± ​0.1^a^	4.3 ​± ​0.3^a^	3.6 ​± ​0.2	2.7 ​± ​0.8^a^	4.5 ​± ​0.1^a^	4.6 ​± ​0.2	4.6 ​± ​0.2	4.5 ​± ​0.2	4.2 ​± ​0.2
	UCO-Nec-1s	3.4 ​± ​0.1	0.2 ​± ​0.1^a^	0.2 ​± ​0.1^a^	3.8 ​± ​0.1	3.6 ​± ​0.2	3.8 ​± ​0.2	4.2 ​± ​0.2	4.3 ​± ​0.2	4.3 ​± ​0.3	4.3 ​± ​0.2	4.1 ​± ​0.3
Lactate (mmol/L)	Sham-UCO	0.9 ​± ​0.1	0.9 ​± ​0.03	0.9 ​± ​0.1	0.9 ​± ​0.0	0.9 ​± ​0.0	1.0 ​± ​0.0	0.9 ​± ​0.1	0.8 ​± ​0.04	0.9 ​± ​0.0	0.8 ​± ​0.0	1.1 ​± ​0.1
	UCO-vehicle	0.8 ​± ​0.1	3.9 ​± ​0.2^a^	6.2 ​± ​0.3^a^	4.8 ​± ​0.7^a^	3.0 ​± ​0.5^a^	2.1 ​± ​0.2^a^	1.5 ​± ​0.2^a^	0.9 ​± ​0.1	0.7 ​± ​0.0	0.8 ​± ​0.0	0.8 ​± ​0.0
	UCO-Nec-1s	0.9 ​± ​0.1	4.7 ​± ​0.3^a^	7.6 ​± ​0.6^a^	5.5 ​± ​0.8^a^	3.3 ​± ​0.7^a^	2.8 ​± ​0.5^a^	1.7 ​± ​0.3^a^	1.0 ​± ​0.1	0.9 ​± ​0.1	0.9 ​± ​0.1	1.0 ​± ​0.1
Glucose (mmol/L)	Sham-UCO	1.1 ​± ​0.1	1.0 ​± ​0.0	1.0 ​± ​0.1	1.2 ​± ​0.1	1.1 ​± ​0.1	1.2 ​± ​0.1	1.1 ​± ​0.1	1.0 ​± ​0.1	1.0 ​± ​0.1	0.8 ​± ​0.1	0.8 ​± ​0.1
	UCO-vehicle	1.0 ​± ​0.1	0.4 ​± ​0.1^a^	0.6 ​± ​0.1^a^	1.3 ​± ​0.1	1.3 ​± ​0.1	1.4 ​± ​0.1	1.4 ​± ​0.1	1.1 ​± ​0.1	0.9 ​± ​0.1	0.9 ​± ​0.1	0.9 ​± ​0.0
	UCO-Nec-1s	1.1 ​± ​0.1	0.3 ​± ​0.1^a^	0.6 ​± ​0.2^a^	1.5 ​± ​0.1	1.4 ​± ​0.1^a^	1.5 ​± ​0.1^a^	1.7 ​± ​0.1^a^	1.3 ​± ​0.1	1.2 ​± ​0.1	1.0 ​± ​0.1	0.9 ​± ​0.1

p_a_CO_2_, arterial partial pressure of carbon dioxide; p_a_O_2_, arterial partial pressure of oxygen; Hct, haematocrit; O_2_ct; arterial oxygen content.

Data are mean ​± ​SEM.

#*p* ​< ​0.05 vs UCO-Vehicle.

a *p* ​< ​0.05 vs. Sham.

In the UCO-Vehicle group, all fetuses reached 25 ​min of occlusion. All but one fetus in the UCO-Nec-1s group reached 25 ​min UCO; in one fetus UCO was stopped at 21:33 ​min because of severe hypotension (MAP <8 ​mmHg). There was no significant difference in area under the curve for MAP spent under 20 ​mmHg between UCO-Nec-1s and UCO-Vehicle groups (4983.6 ​± ​740.4 vs. 4053.8 ​± ​564.6, *p* ​= ​0.374, Tukey post-hoc test). Fetal heart rate and MAP recovered rapidly after the end of occlusion.

Fetal demographics and post-mortem data are shown in Table 2. Heart weight was increased in the UCO-Nec-1s group compared with UCO-Vehicle (*p* ​= ​0.020) and Sham-UCO (*p* ​= ​0.001). The UCO-Nec-1s and UCO-Vehicle groups had smaller lungs than the Sham-UCO group (both *p* ​< ​0.001) but there was no significant difference in lung weight between UCO-Vehicle and UCO-Nec-1s (*p* ​= ​0.382). Brain weight was reduced in the UCO-Vehicle group compared with both Sham-UCO (*p* ​= ​0.003) and UCO-Nec-1s (*p* ​= ​0.041), with no difference between UCO-Nec-1s and Sham-UCO. There were no differences for liver or spleen post-mortem weights between groups.

**Table 2:** Fetal demographics and post-mortem weights.

Group	Sex (F/M)	Body weight (g)	Brain (g)	Lungs (g)
Sham-UCO	4/6	3224.2 ​± ​204.3	39.5 ​± ​0.9	88.9 ​± ​6.4
UCO-vehicle	6/3	3182.2 ​± ​157.4	32.5 ​± ​1.7^a^	44.3 ​± ​5.6^a^
UCO-Nec-1s	2/6	3491.3 ​± ​174.0	37.6 ​± ​1.4^b^	55.2 ​± ​3.3^a^

Group	Heart (g)	Liver (g)	Kidneys (g)	Spleen (g)
Sham-UCO	22.3 ​± ​0.8	112.8 ​± ​10.4	12.4 ​± ​0.7	7.8 ​± ​0.9
UCO-vehicle	20.8 ​± ​0.4	89.9 ​± ​6.8	11.3 ​± ​0.8	6.1 ​± ​0.5
UCO-Nec-1s	26.0 ​± ​1.8^a^^b^	115.2 ​± ​8.1	12.1 ​± ​0.7	7.1 ​± ​0.9

Data are means ​± ​SEM.

a *p* ​< ​0.05 vs Sham-UCO.

b *p* ​< ​0.05 vs UCO-Vehicle.

### White matter injury

As previously reported, marked white matter atrophy and ventriculomegaly developed in 4/9 fetuses in the UCO-Vehicle group, while cystic WMI was observed in an overlapping group of 4/9 fetuses and only 2/9 fetuses did not show either cystic lesions or ventriculomegaly [5]. In the UCO-Nec-1s group, 1/8 fetuses developed focal cystic lesions within the temporal lobe contralateral to the infusion only, 1 fetus had bilateral cystic lesions, white matter atrophy and ventriculomegaly, and 7/8 fetuses had neither cystic lesions nor white matter atrophy nor ventriculomegaly on the side of the infusion (*p* ​= ​0.015 for WMI on the side of the infusion, Fisher Exact test).

**Fig. 2:**
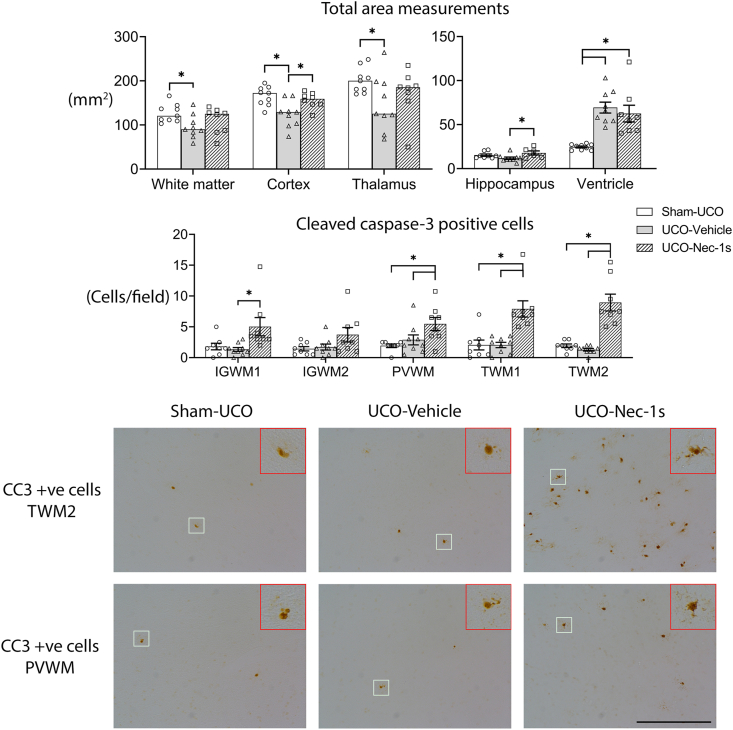
**Total area measurements and apoptotic cell density**. **(Top graphs)** Total area measurements of intact white matter, cortex, thalamus, hippocampus, and ventricles. The UCO-Vehicle group had significantly reduced total intact white matter, cortical and thalamus area compared to the Sham-UCO group. In the UCO-Nec-1s group, there was no significant difference in average white matter and thalamus area compared to either the Sham or UCO-Vehicle group. The cortical and hippocampus area was significantly greater in the UCO-Nec-1s group compared to the UCO-Vehicle group. The UCO-Vehicle and UCO-Nec-1s groups showed a significant increase in ventricle area compared to Sham-UCO. **(Bottom graph)** Shows the cell counts for cleaved caspase-3-positive apoptotic cells. No significant difference was observed between the Sham-UCO and UCO-Vehicle groups in any region. Nec-1s infusion was associated with a significant increase in apoptotic cells in all regions compared to Sham-UCO and UCO-Vehicle groups (∗*p* ​< ​0.05, one-way ANOVA). Data are means ​± ​SEM. **(Bottom images)** Show photomicrographs at 20x magnification of cleaved caspase-3-positive (CC3 +ve) cells with apoptotic features including dense staining and evidence of fragmentation. The top right insets are 3x digital magnifications. Scale bar ​= ​100 ​μm.

Total intact white matter area was reduced after UCO-Vehicle compared with Sham-UCO (Fig. 2, *p* ​= ​0.042). There was no difference in total white matter area measured in the UCO-Nec-1s group compared to either Sham-UCO (*p* ​= ​0.983) or UCO-Vehicle (*p* ​= ​0.090). Further, UCO-Vehicle was associated with reduced total cortical (*p* ​= ​0.007) and thalamic (*p* ​= ​0.042) area compared with Sham-UCO, while there were no differences in cortical, thalamic, and hippocampal area measurements between the Nec-1s group and Sham-UCO. The UCO-Nec-1s group had significantly larger cortical and hippocampal areas compared with the UCO-Vehicle group (*p* ​= ​0.016, *p* ​= ​0.044 respectively). There was increased ventricular area in the UCO-Vehicle and UCO-Nec-1s groups compared with Sham-UCO (both *p* ​< ​0.001) and with no difference between the UCO-Vehicle and UCO-Nec-1s groups (*p* ​= ​0.108).

### Cleaved caspase-3-positive apoptotic cells in white matter regions

Despite evidence of severe macroscopic injury, there was no significant difference in cleaved caspase-3-positive apoptotic cell density between the Sham-UCO and the UCO-Vehicle groups in both the parietal and temporal lobe regions (Fig. 2). Importantly, Nec-1s treatment was associated with increased numbers caspase-3-positive cells showing apoptotic morphology in the parietal IGWM1 and PVWM regions compared to the Sham-UCO (IGWM1, *p* ​= ​0.052; PVWM, *p* ​= ​0.014) and an increase in the IGWM1 regions compared to UCO-Vehicle (*p* ​= ​0.019). A marked increase in apoptosis was also seen in the TWM1 and TWM2 temporal regions of the Nec-1s group compared with both UCO-Vehicle and UCO-Sham groups (all p ​≤ ​0.001).

### Oligodendrocytes and myelin

**Fig. 3:**
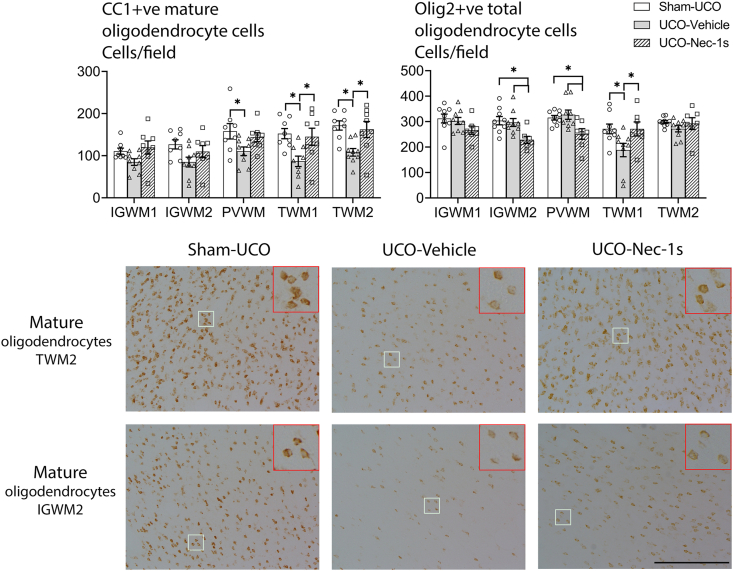
**Nec-1s effects on oligodendrocyte lineages**. **(Left graph)** Shows cell counts for CC1-positive mature oligodendrocytes. UCO-Vehicle was associated with a significant reduction in mature oligodendrocyte density in all regions except IGWM1, while the UCO-Nec-1s group was not significantly different to either Sham-UCO or UCO-Vehicle groups in any region. **(Right graph)** Shows cell counts of labeled Olig2-positive cells, a marker for total oligodendrocytes. Within the parietal lobe regions (IGWM1, IGWM2, PVWM), total oligodendrocyte density was significantly reduced in the UCO-Nec-1s group compared to the Sham-UCO and UCO-Vehicle groups. A significant reduction in total oligodendrocytes was observed in the TWM1 region in the UCO-Vehicle compared to the Sham-UCO groups (∗*p* ​< ​0.05, one-way ANOVA). Data are means ​± ​SEM. **(Bottom images)** Show photomicrographs at 20x magnification of CC1-positive cells in the TWM2 region (top row) and the IGWM2 region (bottom row). The top right insets are 3x digital magnifications. Scale bar ​= ​100 ​μm.

In the IGWM2 and PVWM tracts of the parietal lobe there were fewer Olig2-positive oligodendrocytes (a marker for all oligodendrocyte lineages) in the UCO-Nec-1s group compared to the Sham-UCO (IGWM2, *p* ​= ​0.004; PVWM, *p* ​= ​0.020) and UCO-Vehicle (IGWM2, *p* ​= ​0.017; PVWM, *p* ​= ​0.009) groups. In all parietal lobe regions (IGWM1, IGWM2, PVWM) there was no change in Olig2-positive cell density in the UCO-Vehicle group compared with the Sham-UCO group (Fig. 3). In the TWM1 region of the temporal lobe, there were reduced numbers of Olig2-positive oligodendrocytes in the UCO-Vehicle group compared with the Sham-UCO group (*p* ​= ​0.025), but significantly more Olig2-positive cells in the UCO-Nec-1s group compared with UCO-Vehicle (*p* ​= ​0.025), to levels similar to the Sham-OCU group (*p* ​= ​0.977). There were no differences between any groups in the TWM2 region.

There was a significant reduction in CC1-positive mature oligodendrocytes in the UCO-Vehicle group compared with the Sham-UCO group in the PVWM, TWM1, and TWM2 (*p* ​= ​0.046, *p* ​= ​0.015, *p* ​= ​0.007 respectively; Fig. 3), and a trend toward reduced numbers in the IGWM2 region (*p* ​= ​0.059), TWM1 *p* ​= ​0.006, TWM2 *p* ​= ​0.003). In the parietal regions, the UCO-Nec-1s group showed intermediate numbers of CC1-positive cells, which were similar to both the Sham-UCO (IGWM1 *p* ​= ​0.836, IGWM2 *p* ​= ​0.605, PVWM *p* ​= ​0.744) and UCO-Vehicle (IGWM1 *p* ​= ​0.075, IGWM2 *p* ​= ​0.340, PVWM *p* ​= ​0.195) groups. However, in the temporal regions, there was a significant increase in numbers of CC1-positive cells in the Nec-1s group compared with the UCO-vehicle group (TWM1 *p* ​= ​0.032, TWM2 *p* ​= ​0.023), to levels similar to the Sham-UCO group (TWM1 *p* ​= ​0.939, TWM2 *p* ​= ​0.873).

**Fig. 4:**
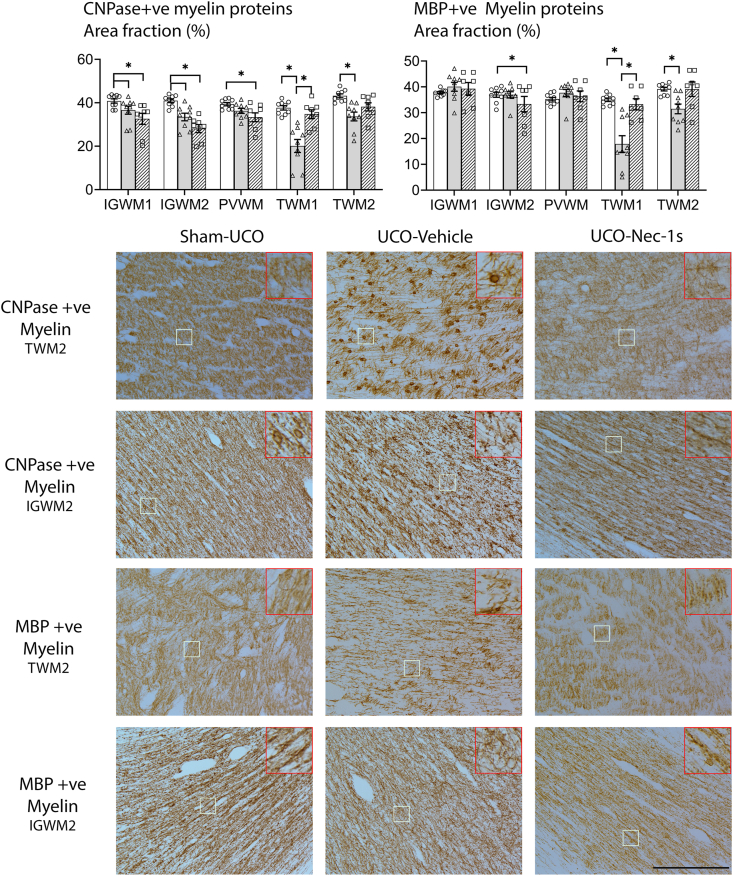
**Nec-1s effects on myelination**. **(Left graph**) Shows area fraction results of CNPase-positive myelin. The UCO-Nec-1s group had a significantly lower CNPase-positive area fraction compared to Sham-UCO in all parietal regions, but no difference was observed in the temporal lobe regions. A significant reduction in CNPase-positive area fraction was observed in the UCO-Vehicle group compared to the Sham-UCO group within both the temporal lobe regions. (**Right graph**) Shows area fraction results of MBP-positive myelin. A significant reduction was observed in the IGWM2 region between the UCO-Nec-1s group compared to both the Sham-UCO and UCO-Vehicle groups. In the temporal lobe, the UCO-Vehicle group had a significantly lower MBP area fraction compared to Sham-UCO, while the UCO-Nec-1s group was not different from Sham-UCO (∗*p* ​< ​0.05, one-way ANOVA). Data are means ​± ​SEM. **(Bottom images)** Show photomicrographs of CNPase and MBP-positive myelin protein at 20x magnification in the TWM2 and the IGWM2 regions. The top right insets are 3x digital magnifications. Scale bar ​= ​100 ​μm.

Reduced expression of the myelin components CNPase and MBP was observed in the UCO-Vehicle group (Fig. 4), with reduced CNPase-positive area fraction compared with the Sham-UCO group in the parietal IGWM1 (*p* ​= ​0.007) and IGWM2 region (*p* ​= ​0.002), and within and around the temporal lobe (TWM1, *p* ​< ​0.001; TWM2, *p* ​< ​0.001). There was also a significant reduction in the MBP-positive expression in the UCO-Vehicle group compared with the Sham-UCO group in the TWM1 (*p* ​< ​0.001) and TWM2 (*p* ​= ​0.002) regions. The UCO-Nec-1s group showed a significant reduction in CNPase-positive area fraction in the parietal lobe regions (IGWM1, *p* ​= ​0.007; IGWM2, *p* ​< ​0.001; PVWM, *p* ​= ​0.007), and reduced MBP-positive area fraction in the IGWM2 (*p* ​= ​0.009) region, compared with the Sham-UCO group, with no differences compared with UCO-Vehicle. Further, the UCO-Nec-1s group showed a trend toward a reduction in CNPase-positive area fraction in the IGWM2 region compared with the UCO-Vehicle group (*p* ​= ​0.053). In the temporal lobe, there were no differences in the CNPase-positive area fraction between the UCO-Nec-1s and Sham-UCO groups in the TWM1 (*p* ​= ​0.179) and TWM2 (*p* ​= ​0.154) regions. Similarly, there were no differences in the MBP-positive area fraction between the UCO-Nec-1s and the Sham-UCO groups in the TWM1 (*p* ​= ​0.432) and TWM2 (*p* ​= ​0.382) regions.

### Microglia and astrocytes

**Fig. 5:**
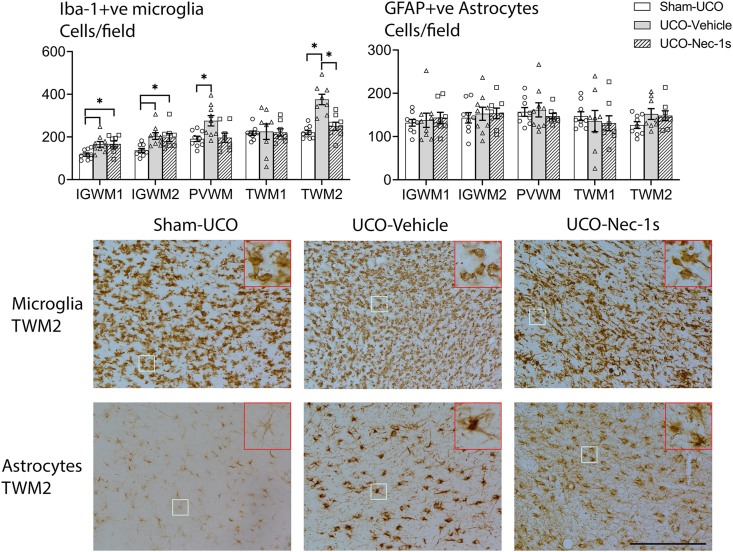
**Microglia and astrocyte cell densities**. **(Left graph**) Shows cell counts for Iba-1-positive microglia. The UCO-Vehicle group had increased numbers of microglia compared to Sham-UCO in all regions, except the TWM1 region. The UCO-Nec-1s group also showed a significant increase in microglia in the IGWM1 and IGWM2 regions compared to Sham-UCO. In the TWM2 region, UCO-Nec-1s was associated with reduced microglia compared with the UCO-Vehicle group but not compared to Sham-UCO. (**Right graph**) Shows cell counts for GFAP-positive astrocytes. No significant difference in astrocyte cell density was observed between any group in any region (∗*p* ​< ​0.05, one-way ANOVA). Data are means ​± ​SEM. **(Bottom images)** Show photomicrographs at 20x magnification of Iba-1-positive microglia (top row) and GFAP-positive astrocytes (bottom row) in the TWM2 region. The top right insets are 3x digital magnifications. Scale bar ​= ​100 ​μm.

Iba-1-positive microglia density was increased in the UCO-Vehicle group compared with the Sham-UCO group in the IGWM1 (*p* ​= ​0.021), IGWM2 (*p* ​= ​0.008), PVWM (*p* ​= ​0.015), and TWM2 (*p* ​< ​0.001) (Fig. 5). Qualitatively, Iba-1 positive cells in the TWM1 region were larger and more spread out. The UCO-Nec-1s group also showed a significant increase in microglial density in the IGWM1 (*p* ​= ​0.020) and IGWM2 (*p* ​= ​0.023) regions, but no difference in the PVWM region (*p* ​= ​0.981), compared with the Sham-UCO group. In the TWM2 region, there was a significant reduction in microglial density in the Nec-1s group with compared with the UCO-Vehicle (*p* ​< ​0.001), to levels similar to the Sham-UCO group (*p* ​= ​0.498). There were no differences in microglial density between groups in the TWM1 regions. Further, there was no significant differences in GFAP-positive astrocyte cell density between any group in any region (Fig. 5).

## Discussion

In the present study, delayed ICV infusions of Nec-1s starting 72 ​h after HI were associated with reduced risk of severe WMI in the hemisphere on the side of the infusion, with an overall reduction in the severity of WMI in the temporal lobe and improved cortical and hippocampal areas after 3 weeks of recovery. These data strongly support the hypothesis that late-developing severe WMI is in large part mediated by necroptosis. Interestingly, inhibition of necroptosis was associated with upregulation of cleaved caspase-3-positive apoptosis in both the temporal and parietal lobes, suggesting that the cell death pathway was redirected toward apoptosis. The reduced density of microglia after Nec-1s infusion suggests that apoptosis is less inflammatory. Speculatively, this may disrupt the inflammatory feedback loop associated with necrosis, leading to a net reduction in total inflammatory load within the brain. Taken with our previous finding of improved WMI after Etanercept infusion [6], the present data support the potential for benefit with even delayed anti-inflammatory/anti-necroptosis interventions.

HI induced by 25 ​min of UCO was associated with severe WMI, including cystic lesions with loss of all cells in the center, surrounded by astrogliosis and microglial infiltration [23], white matter atrophy and ventriculomegaly. Diffuse WMI was also observed around the periphery of the cystic WMI in the temporal lobe, as shown by significant loss of myelin components and reduced density of mature oligodendrocytes (i.e. dysmaturation), without areas of pan-cell loss [24]. Microcysts were not seen. Importantly, the development of cystic WMI by 21 days recovery is consistent with the clinical pattern of severe injury seen on serial cranial ultrasounds from 3 to 5 weeks after birth in extremely preterm infants [3,25]. Previously, it was believed that the majority of cell death is established by 72 ​h after perinatal HI, corresponding to the end of the period of secondary metabolic failure, seizures and widespread cell death known as the secondary phase [5], and thus that treatment after this time would be limited to supporting neurorepair and reorganisation of the remaining neural networks rather than preventing bulk cell death. The present proof of principle study, in conjunction with previous studies [5,6], suggests that there may be a much wider window of opportunity to prevent delayed WMI mediated by necroptosis in the preterm-equivalent brain.

The present study demonstrates that Nec-1s significantly ameliorated diffuse WMI and markedly improved expression of myelin components and numbers of mature oligodendrocytes in the temporal lobe, although interestingly, not in the parietal lobe. There is evidence that if one cell death pathway is blocked, alternative pathways may still be activated [8]. For example, inhibition of apoptosis by caspase-8 blockade was associated with greater necroptosis [26]. If this is correct, this suggests that blockade of necroptosis death pathways after RIP1 recruitment may have promoted the progression through extrinsic apoptotic pathways [27]. In the present study, Nec-1s administration appeared to have exacerbated the loss of total oligodendrocytes labeled with Olig2 as well as reduced CNPase and MBP-positive expression within the parietal lobe white matter, suggesting there are region-specific differences (Fig. 3, Fig. 4). In cell culture, Nec-1 exposure reduced cellular FLISS inhibitory protein (cFLIP) which is a potent anti-apoptotic gene [28]. As shown previously [5], the parietal lobe can recover to near sham levels after severe HI with no intervention, raising the possibility that disruption of cFLIP gene expression after UCO-Nec-1s may have increased pro-apoptotic signaling in cells in the parietal lobe that would have otherwise survived (Fig. 2).

By contrast, there was overall improvement in the temporal lobe after delayed Nec-1s infusion. In this region, a shift in the predominant cell death pathway away from necroptosis toward apoptosis may have attenuated the severity of neuroinflammation. Chronic inflammation is a key feature of the tertiary phase that can promote necroptosis [[29], [30], [31]]. Because Iba-1 labels almost all cells in the microglial/monocyte lineage, including resident microglia and peripheral monocytes, elevated Iba-1+ cell counts can include infiltration from the periphery. Interestingly, in neonatal HI models, inflammatory monocyte entry is temporally phased, with biphasic peaks around 1 day and 7 days post-injury [32,33]. Further, necroptosis is itself pro-inflammatory, as cell membrane rupture releases pro-inflammatory cytokines and debris into the extracellular space, which induces a microglial response [34]. In turn, as seen in human neonates, activated microglia are associated with a local increase in pro-inflammatory cytokines, such as TNF, and with WMI [35,36]. In support of this hypothesis, in the present study, UCO was associated with increased numbers of microglia, which was attenuated in the temporal lobe by Nec-1s treatment (Fig. 5). This is consistent with findings from other studies investigating the neuroprotective effects of RIP1 blockade in rodent models of traumatic brain injury and in models of neonatal HI [8,[37], [38], [39]]. For example, in a Rice Vannucci mouse model of HI at postnatal day 7 (P7), Northington et al. reported that Nec-1 infused 15 ​min after HI significantly decreased TNF levels and reduced Interleukin (IL)-1β, IL-6, and IL-12 protein markers within 24 ​h [8].

In preterm fetal sheep, we have previously reported reduced cortical area and perimeter but maintained neuronal density after 3 weeks recovery from 25 ​min of UCO. This reduction was potentially mediated by reduced dendritic arborisation and synaptic density in cortical projection neurons [40,41]. It will be important in future studies to test whether interventions such as Nec-1s can mitigate these cortical alterations and preserve connectivity. Encouragingly, in the present study, infusion of Nec-1s was associated with recovery of both total cortical and hippocampal area to Sham-UCO values (Fig. 2). Potentially, these beneficial effects of RIP1 blockade on gray matter regions may be mediated by both indirect and direct effects. Inflammation can disrupt normal astrocyte function, which likely impedes the development of projection fibers to and from the cortex thus impairing cortical growth [42]. Although cortical inflammation after Nec-1s treatment requires further investigation, Nec-1s may have indirectly improved cortical maturation by reducing neuroinflammation in the temporal lobe (Fig. 5) which allowed improved cortical recovery. Alternatively, Nec-1 given 15 ​min after HI in the P7 Rice–Vannucci mouse model reduced mitochondrial dysfunction, nitric oxide accumulation and glutathione oxidation of cortical neurons after 24 ​h recovery [14]. Further, in the same model, Nec-1 treatment ameliorated neuronal endoplasmic reticulum dysfunction typically observed 24 ​h after HI [43]. Although these studies focused on the effects of early treatment and concentrated on changes in the secondary phase of injury, RIP-1 inhibition may have both early and delayed effects by attenuating inflammation and reducing NO release, which protects mitochondria and reduces subsequent cell death [14].

Notably, Northington and colleagues observed potential sex differences in the protective effects of Nec-1 treatment [8]. In an *in-vivo* study, Nec-1 inhibited translocation of apoptosis-inducing factor, which in rodents is more expressed in males than females following HI [44,45]. Given that inflammation, oxidative stress, and RIP1-RIP3 interaction precede the translocation of apoptosis-inducing factor, it is plausible that the reduction of these factors after Nec-1 administration may have contributed to the greater neuroprotection observed in male rodents [8]. The present study was unfortunately not powered to examine sex differences, and a much larger study will be needed to examine this possibility.

### Perspectives and significance

Similarly to other preclinical models, this preparation does not incorporate the effects of postnatal physiology, sensitizing events before or after HI, or the influence of other obstetric conditions such as intrauterine growth restriction, preeclampsia, and infection, all of which can affect brain development and outcomes in preterm infants [46]. Further, gray matter injury is an important part of the spectrum of brain injury seen in preterm infants with severe cystic necrotic WMI [47,48], and it will be important to investigate the impact of Nec-1s in future studies.

The reader should note that many outcomes after Nec-1s infusion were intermediate between the UCO-Vehicle and the treatment group. This is likely due to relatively low number of animals and/or that alterations to the dosage and timing of treatment would be needed for optimal outcomes. Further, more research is needed to investigate potential sex differences in the neuroprotective effects of Nec-1s, and to explore the potential regional differences in the response to treatment. A further limitation of the present study is that the evidence behind necroptosis is based on the observations of similar studies, as well as the low expression of apoptosis markers. Currently, no immunohistochemistry marker is available to identify cells undergoing necrosis in paraffin embedded tissue. Electron microscopy is the gold standard method to confirm necrosis; however, the specific tissue preparation needed is incompatible with the tissue preparation used in this study. Moreover, severe necrosis leads to a profound loss of all glial elements such that the cystic lesions typically lack residual tissue for histological analysis. Therefore, further analysis using electron microscopy is needed to confirm the presence of necrosis in the UCO-Vehicle group, as well as to confirm the reduction in necrosis in the UCO-Nec-1s group.

In conclusion, the present study provides further evidence that delayed, severe WMI is mediated by slowly evolving necroptosis and neuroinflammation. In preterm fetal sheep exposed to severe HI, inhibition of necroptosis with Nec-1s was associated with reduced risk of severe WMI in the temporal lobe on the side of the infusion and reduced inflammation with improved numbers of oligodendrocytes and myelin components. Importantly, protection was specific to the temporal lobe compared with the parietal lobe, suggesting that further studies of the longer-term impact of Nec-1s and similar agents will be needed. Overall, these results suggest that necroptosis is a promising therapeutic target to mitigate severe white matter injury.

## Author Contribution

BAL, LB and AJG conceptualized this study. BAL, CAL, SKD, VJK, JMD and JOD undertook experiments, histological and data analysis, and prepared figures. BL wrote the first draft of the manuscript. All authors critically revised the manuscript and approved the final manuscript as submitted and agree to be accountable for all aspects of the work.

## Declaration of competing interest

The authors declare the following financial interests/personal relationships which may be considered as potential competing interests: Alistair Jan Gunn reports financial support was provided by the Health Research Council of New Zealand (grant 22/559). The other authors declare that they have no known competing financial interests or personal relationships that could have appeared to influence the work reported in this paper.
